# A Study of Transmigrated Canine in an Indian Population

**DOI:** 10.1155/2014/756516

**Published:** 2014-11-10

**Authors:** Gaurav Sharma, Archna Nagpal

**Affiliations:** ^1^Department of Oral Medicine and Radiology, Sudha Rustagi College of Dental Science and Research, Faridabad, Haryana 121002, India; ^2^Department of Oral Medicine and Radiology, PDM Dental College & Research Institute, Bahadurgarh, Haryana 124507, India

## Abstract

*Aim*. The purpose of this study was to investigate the prevalence of transmigrated canines in a north Indian population and association with gender, side, associated pathologies, and dental anomalies. *Subjects and methods*. The prospective study consisted of panoramic radiographs of 3000 patients from two dental colleges in north India. The panoramic radiographs were screened for radiographically identified position of the transmigrated tooth, retained canine, and other coexisting dental anomalies. *Results*. The overall prevalence of transmigrated canines (15 mandibular and 5 maxillary) was 0.66%. The prevalence of mandibular transmigrated canine was 0.5% and maxillary transmigrated canine was 0.16%. All the transmigrated canines were unilateral. The age range was 15–53 years (average age 24.1 years) and there were 12 males (60%) and 8 females (40%). Type 1 mandibular canine transmigration was the commonest type found in our study (10 cases), followed by types 2 and 4 (2 cases each) and 1 case of type 5 transmigration. *Conclusion*. The prevalence of transmigrated canines in the north Indian population was 0.66% and no gender predilection was evident. The transmigrated canines have a low complication rate (10.0%) and no correlation with other dental anomalies was found. Type 3 canine is the rarest form of mandibular canine transmigration.

## 1. Introduction

The preeruptive migration of a tooth across the midline is termed as “transmigration.” Initially, the term transmigration was used only if the entire length of the impacted canine had migrated and crossed the midline of the mandible [1]. However, Javid modified the definition by suggesting that one half or more of the length of the tooth was required to cross the midline in order to be considered as transmigration [2, 3]. Joshi suggested that rather than the distance of migration, the tendency of a canine to cross the barrier of the midline suture was more important [4]. Though a number of factors have been proposed, the aetiology and exact process of transmigration are indeterminate [3]. The prevalence of transmigration in different populations and ethnic groups was the subject of several studies and was reported to be between 0.1 and 0.34% [5–7]. In India only one study has been conducted on transmigrated mandibular canines [8]. To the best of our knowledge, the present study is the first to report the prevalence of both transmigrated maxillary and mandibular canine in India. In the current study, we determine the prevalence of transmigrant maxillary and mandibular canines in a north Indian population and describe the clinical and radiographic features of patients with transmigrant impacted canines.

## 2. Subjects and Methods

A prospective study of 3000 panoramic radiographs (1695 males and 1305 females) was conducted in two dental colleges in Haryana for 8 months from September 2013 to April 2014. All the digital panoramic radiographs were evaluated. The radiographs of completely edentulous patients and paediatric patients below the age of 10 years were excluded. The radiographs were screened for transmigrant canines by a single examiner experienced in dentomaxillofacial radiology. The examiner in the study was blinded to patient data during the radiographic examination procedure. An impacted canine was considered transmigrated when the tip of the crown of the canine regardless of its length had crossed the midline [4]. The panoramic radiographs were screened for radiographically identified position of the transmigrated tooth, retained canine, associated pathologies, and other coexisting dental anomalies (other than third molar). All digital images were stored in a computer database using the manufacturer's software. Each image's contrast and brightness were optimized to produce the best image for viewing under standardized conditions. After diagnosing a transmigrant canine radiographically, clinical evaluation was done for the patient's age and sex, symptoms, the presence of associated pathology, and the treatment provided. As diagnosis of transmigrated canine is an objective assessment and these teeth are clearly visible there was no requirement for an interexaminer validity. Statistical analysis was done using SPSS version 11 and the Chi-square test was done. The level of significance was set to *P* < 0.05.

The transmigrant mandibular canines were classified according to Mupparapu [9]. The classification can be summarized as follows.

**Type 1 .**

Canine positioned mesioangularly across the midline, labial or lingual to the anterior teeth.

**Type 2 .**

Canine horizontally impacted near the inferior border of the mandible inferior to the apices of the incisor teeth.

**Type 3 .**

Canine erupting on the contralateral side.

**Type 4 .**

Canine horizontally impacted near the inferior border of the mandible below the apices of posterior teeth.

**Type 5 .**

Canine positioned vertically in the midline with the long axis of the tooth crossing the midline.

The relationship (position/angulation) of maxillary transmigrant canine with median palatine suture and the distance travelled in contralateral arch past midline suture by transmigrated canine along its long axis was also evaluated. The maxillary transmigrated canine was approximated into three parts along its long axis as tip of crown till approximate halfway of crown, halfway of crown till cement-enamel junction (CEJ), and lastly beyond CEJ irrespective of root length of transmigrated canine (Figure 7).

## 3. Results

**Figure 1:**
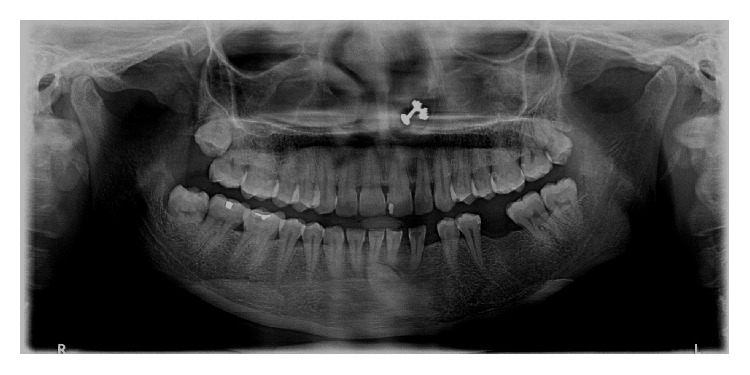
Panoramic radiograph depicting transmigrant mandibular left canine (33) showing Type 1 transmigratory pattern (case 1).

**Figure 2:**
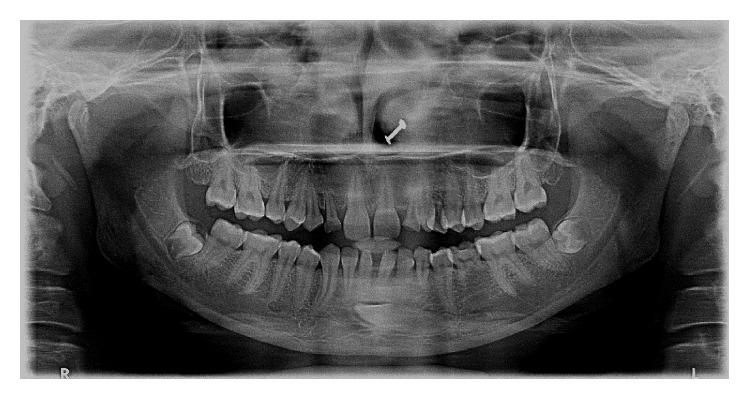
Panoramic radiograph depicting transmigrant mandibular left canine showing Type 2 transmigratory pattern (case 4) with retained deciduous maxillary canines and left mandibular second molar with agenesis of maxillary right canine and mandibular left second premolar. (Microdont maxillary lateral incisors are also visible.)

**Figure 3:**
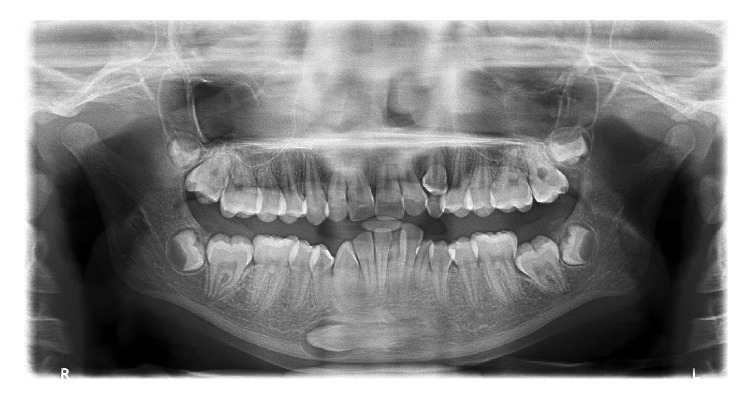
Panoramic radiograph depicting transmigrant mandibular left canine with enlarged follicle space showing Type 4 transmigration pattern (case 16). Overretained left maxillary and mandibular canines are present.

**Figure 4:**
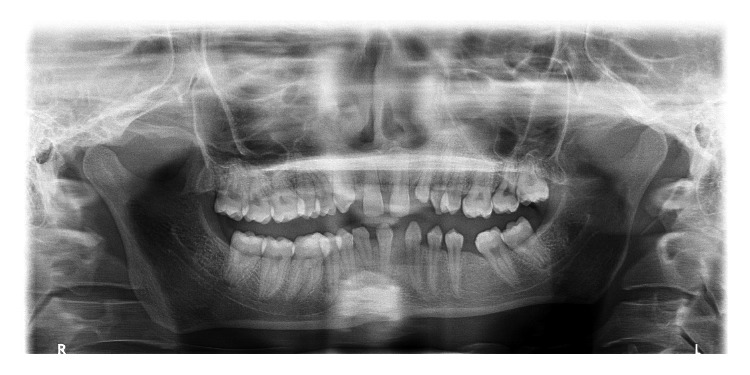
Panoramic radiograph depicting transmigrant mandibular right canine showing Type 5 transmigration (case 6) with agenesis of mandibular left incisors and right lateral incisor.

**Figure 5:**
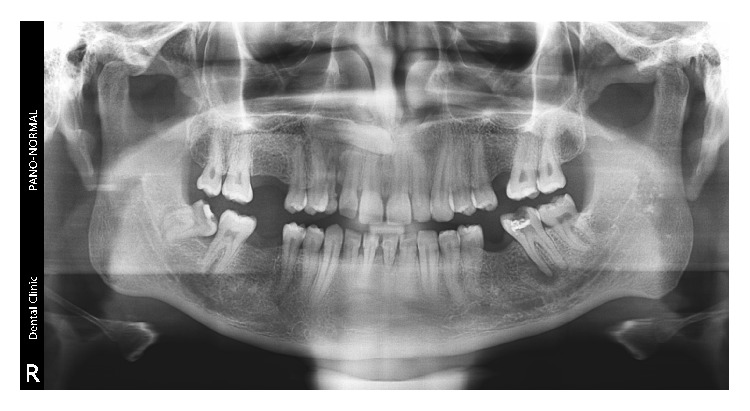
Panoramic radiograph depicting transmigrant maxillary right canine (case 20) with overretained deciduous right maxillary canine.

**Figure 6:**
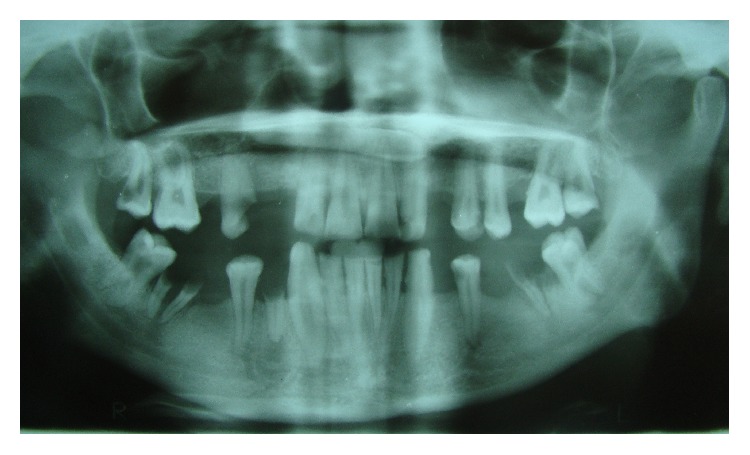
Panoramic radiograph depicting transmigrant maxillary right canine (case 11).

**Figure 7:**
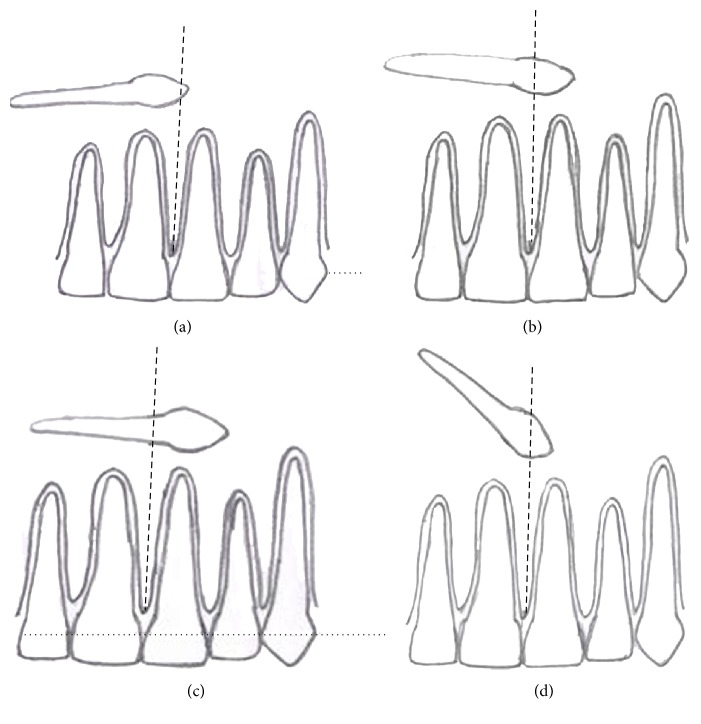
An illustrative depiction for the evaluation of position/angulation of transmigrant maxillary canine with median palatine suture. (a) Contralateral arch distance travelled past midline suture by horizontally placed transmigrated canine along its long axis with tip of crown till less than halfway length of crown. (b) Distance travelled for halfway of crown till cementoenamel junction of horizontally transmigrated canine. (c) Distance travelled beyond cementoenamel junction irrespective of length of root of horizontally transmigrated canine. (d) A mesioangularly impacted transmigrated maxillary canine (45°) with tip of crown till less than halfway length of crown of transmigrated canine.

There were 20 patients (12 males and 8 females) observed in our study with transmigrated canines. The mean age of patients with transmigrated canines was found to be 24.1 years (age range 15–50 years). The overall prevalence of transmigration (maxillary and mandibular) was 0.66%. The prevalence of mandibular canine transmigration was 0.5% (15/3000) whereas the prevalence of 0.16% (5/3000) was recorded for the maxillary canine transmigration (*P* < 0.05) (Figures 1, 2, 3, 4, 5, and 6). The results are summarized in Table 1. There was no statistically significant difference for gender with 12 transmigrant mandibular canines in males (0.7%) as compared to 8 subjects with transmigrant canines (0.61%). All the transmigrated canines were unilateral. Left-side predominance was observed for mandibular canine transmigration with 11 patients observing left mandibular canine migrating towards right side and four cases migrating from the right side towards left side (*P* < 0.5). A similar finding was observed in maxillary canine transmigration with 3 cases depicting a left maxillary canine migration as compared to two cases depicting right side transmigration. There was presence of the retained primary canine with respect to transmigrated canine in seven cases (35.0%). The presence of other dental anomalies was present in 45% cases of transmigration (*P* > 0.05). Applying Mupparapu's classification for mandibular canine transmigration (15 cases), Type 1 transmigration was the commonest type found in our study (10 cases), followed by Types 2 and 4 (2 cases each) and 1 case of Type 5 transmigration. No Type 3 transmigration case was found in the study. 2 cases (10.0%) of transmigrated canines had enlarged follicular spaces. All the patient's clinical and radiological features are summarized in Table 2. Only the tip of crown till approximate half of crown positioned in the median palatine suture was observed in 60% cases of transmigrant maxillary canines (Figure 7). Four maxillary transmigrant canines were positioned in a horizontal or perpendicular direction (80°–90°) to median palatine suture as compared to single case with mesioangular impaction (Table 3).

## 4. Discussion

In the present study, the prevalence of maxillary and mandibular canine transmigrated canines was evaluated which is in contrast to the only previous Indian study related to transmigrated teeth, that focussed only on mandibular canines [8]. Transmigration, intraosseous migration of unerupted tooth, is a rare phenomenon [5, 6]. Early diagnosis with a timely orthodontic or surgical intervention can help dentists preserve the canines, which play an important role, in both aesthetics and function in the human dentition. The research on prevalence of transmigrated canines has been scarce with only a handful of studies conducted on this rare phenomenon (Table 4) [5–16].

In accordance with the previous studies, a greater predilection of mandibular canine transmigration (0.5%) was observed in our study as compared to maxillary canine transmigration (0.16%). Aktan et al. had observed a similar predilection of  0.14% for maxillary transmigrated canines and found a prevalence of 0.34% for transmigrated mandibular canines, while Gunduz found a 0.1% prevalence of transmigrated canines in 12,000 patients [6, 7]. The prevalence of transmigrated maxillary canine (0.16%) found in our study was found to be similar to all the studies, though a greater prevalence of 0.44% was documented in one study [5]. The prevalence of transmigrated mandibular canine was, however, found to be variable ranging from 0.004% by Mupparapu [9] to 0.46% by Kumar et al. [8]. However, the overall prevalence of transmigration of 0.66% was found to be slightly higher than the other studies. Zvolanek's case series failed to find any transmigrant canines in 4000 individuals [17] whereas Javid had identified one transmigrant canine after examining 1,000 panoramic radiographs [2]. The difference in prevalence can be attributed not only to ethnic differences but also to type of the study population whether it is orthodontic or general population.

Similar to the findings reported by Aydin et al. [10], we also found that transmigrant canines occur more frequently in male patients (60%) than in female patients (40%). This finding was in contrast to a study conducted by Halcioglu et al. and González-Sánchez et al. who found a greater prevalence of transmigrated canines in females [15, 18]. In an extensive review by Sumer et al. they had postulated a slight female predilection (1.6 : 1) [19]. Aras et al. had, however, observed no gender predilection in their study of 6000 patients [12]. The reason for the gender predilection is not clear, though females typically tend to report frequently for aesthetics as compared to males [6]. In our study there was a marginally increased number of males (1695 males; 1305 females) that possibly could have resulted in greater number of transmigrated canines being observed in males, though no statistically significant difference was found (*P* > 0.05).

The reported patient age at presentation of the transmigrant canine varies from 8 years to 69 years [17]. In our study the average age was found to be 24.1 years with age range of 15–53 years. The mean age for maxillary transmigrant canines (32 years) was found to be much higher as compared to transmigrant mandibular canine (21.5 years). This increased mean age could be attributed to a greater path travelled as well as median palatine barrier that has to be crossed by maxillary canine to transmigrate as compared to relatively easier path for mandibular canine. Nevertheless, this finding could be coincidental and more studies regarding transmigrant maxillary canine can prove that whether transmigrant maxillary canine is likely to be encountered in a slightly higher age group as compared to mandibular canine. Moreover, the lack of previous radiographic records further makes this finding difficult to ascertain.

All the transmigrant cases reported in present study were unilateral. Canine transmigration involved a left-side tooth more often (14/20 cases; 70%) than the right canine (6/20; 30%). This finding was in accordance with the findings observed by Kumar from South India, Celikoglu from Turkey, and Mazinis in Greece [8, 13, 16]. However Aydin et al. and Aras et al. had not found any side predilection in their study [10, 12]. In the 28 cases documented by Joshi, though no prevalence was reported, 53.6% were left-side teeth, 32.1% were right-side ones, and 14.6% were bilateral cases [4]. Bilateral canine transmigrations are rare and very few case reports have been documented in literature [20–24]. Joshi et al. was the first to report the bilateral occurrence of transmigrated canines and later Mupparapu et al. had also postulated intraosseous pattern of the bilateral transmigration of canines [24]. All documented bilateral transmigrant canine cases have involved mandibular canines except a single reported case of bilateral maxillary transmigrated canines in study conducted by Aktan et al. [6] The findings in the current study did not reveal any case of bilateral transmigration.

Despite the first case of transmigration reported by Caldwell in 1955, the etiology of transmigration is still an enigma [25]. A possible etiological factor of transmigration postulated was retention or premature loss of deciduous teeth [18]. 35% of the cases in our study had retained primary canine with respect to the transmigrated tooth. Other factors postulated were crowding, spacing, supernumerary teeth, unfavourable alveolar arch length, fractures with displacement of tooth bud, and an excessive crown length of the permanent canine [9, 18]. Crowding of the mandibular incisors was present in only one case (5.0%) in our study. Joshi had stated that the presence of cyst, tumor, or odontoma may facilitate transmigration of the canine [4]. In the current study, no maxillary or mandibular canine transmigrations were associated with any pathological entities. Aras et al. evaluated that impacted transmigrant canines were not associated with any pathology in 6,000 patients [12]. Celikoglu had also observed similar findings of no pathological entities. Bruzst suggested that the canine tooth bud is situated in front of the lower incisors and that facial growth pushes the canine towards the contralateral side [26]. However, one possible theory that has been postulated is heredity [27]. Recently, Kontham et al. had documented transmigration of mandibular canines in siblings [28]. The future studies of transmigration should focus also on the patient's siblings and parents for the possible hereditary pattern.

Pippi and kaitsas had postulated a pericoronal osteolytic area, because of an anomalous secretion of signal molecules either due to genetic origin or inflammatory stimuli [3]. Therefore, a symmetrical pericoronal enlargement takes place that represents a “locus minoris resistentiae,” towards which the impacted canine moves. This movement stops when the tooth finds a mechanical obstacle (e.g., jaw cortical bone). Vichi and Franchi had suggested that agenesis of the adjacent teeth, in particular, the lateral incisor, may favour retention of the primary canine, and the excess of space in the dental arch may account for the absence of a correct guide for eruption [29]. Though there were 2 concurrent cases of agenesis of maxillary lateral incisors and one case of congenitally missing maxillary canine with mandibular canine transmigration, none of these cases were sufficient to warrant a statistical analysis. The authors correlated the association of transmigration with other dental anomalies (other than third molars) but could not find any significant finding. The rarity of transmigrant canines in our study and other published studies in literature makes the correlation studies difficult. The reason why an impacted canine transmigrates might be due to concoction of all the above factors that could possibly lead to varying presentations of particular transmigrant tooth.

The likely reasons for a greater number of mandibular transmigrant cases are voluminous mandibular symphysis and an emphasised buccal inclination of the lower incisors, as well as the typical conic shape of the canine root and crown [3]. Transmigration of an impacted maxillary canine is rare due to the negligible distance between the apexes of the maxillary incisors and the floor of the nasal fossae and to the presence of the midpalatal suture, which is a considerable barrier against maxillary canine migration [10]. In three cases (60%), tip of crown till less than halfway of crown of transmigrant maxillary canines was positioned in the median palatine suture whereas in 40% of the cases more than half of crown (till cementoenamel junction) of transmigrant maxillary canine was observed to cross the median palatine barrier. The above result is in agreement with other studies where crown of transmigrant maxillary canines could traverse only till cement-enamel junction beyond median palatine suture and no case has yet been reported where a maxillary canine has completely crossed into the contralateral arch [6, 11, 16]. Currently it is not known how much eruptive force is required but the transmigrant maxillary canine might be having a greater amount of horizontal component of eruptive force to penetrate the resistance of dense median palatine suture [30].

The type of the angulation formed between the midsagittal plane and unerupted canine decides the course of transmigration [10]. It has been suggested that when the angle exceeds 50°, mandibular canines are likely to migrate and cross the midline [10]. However if the angulation is less than 30°, transmigration is unlikely. Though radiographic technique dictates the appearance of radiographic position of transmigrated maxillary canine, a horizontal position (80°–90°) of transmigrant maxillary canine was observed in four cases, whereas mesioangular position (45°) was observed in one case. Shapira and Kuftinec had suggested that the migrating maxillary canine probably tunnels through the bone as median palatine suture typically remains intact radiographically in all previously documented cases [31]. Thus a higher angulation (approximately 80°) as compared to mandibular canine might be required for an impacted maxillary canine to overcome strong anatomical barrier of median palatine suture and hence a horizontal position is more likely to transmigrate as compared to a mesioangular position. However more studies are required to corroborate the above finding of angulation and position making a difference in penetration of suture as there is still paucity of reported cases of maxillary transmigrant canines.

According to Mupparapu the most likely position of the transmigrant mandibular canine is mesioangular impaction (Type 1) and the erupted transmigrant canines especially in vertical position in midline are the rarest (Type 5) [9]. In our study, Type 1 transmigration was the pattern most frequently encountered in accordance with the previous studies. No Type 3 transmigration was observed in our study. These findings were similar to the studies conducted by Kumar et al, Mazinis et al., and Aktan et al. [6, 8, 16] The Type 3 canine transmigration appears to be the rarest form of mandibular transmigration rather than Type 5 after reviewing all the published studies on transmigration. The classification by Mupparapu (2002) could not be used for maxillary transmigrated teeth [6], though there was mild variation in the patterns of transmigrated maxillary canines in position and angulation.

The transmigrated canines are mostly asymptomatic, though, rarely complications like follicular space enlargement, chronic infection with fistula, pain due to impingement, resorption of adjacent teeth, and swelling as well as ectopic eruption could occur. In our study we observed two cases (10.5%) of transmigrant canine with follicular space enlargement. None of the patients in the present study were symptomatic and no root resorption was evident in adjoining teeth of the transmigrant canines. Mazinis et al. had however observed a single case of root resorption in lateral incisor and had postulated usage of cone beam computed tomography (CBCT) for accurate localisation and assessing the root resorption [16].

Proposed treatment strategies for transmigrated mandibular canines include surgical removal, transplantation, and surgical exposure with orthodontic alignment. The most preferred treatment for migrated canines is surgical extraction [8, 18]. This is especially true when the mandibular arch is crowded and requires therapeutic extractions to correct the incisor crowding. It is however imperative to anesthetise the nerve on the originating side as transmigrated teeth maintain their nerve connection to the originating side where the tooth germ is formed [4]. If the mandibular incisors are in normal position with sufficient space for transmigrated canine, then transplantation can be undertaken [32].

Wertz stated that if the crown of a transmigrated tooth migrates past the opposite incisor area, or if the apex migrates past the apex of the adjacent lateral incisor, it might be mechanically impossible to bring it into place [33]. Alaejos-Algarra et al. had suggested that when the transmigrated canine is accessible, and especially if it is symptomatic, removal of the impacted tooth is recommended [34]. If asymptomatic, transmigrant canine should be periodically evaluated radiographically and kept under observation [34]. Since none of our cases were symptomatic, removal of impacted transmigrated tooth was not considered. All the patients were kept on periodic follow-up and referred for orthodontic treatment with varying management modalities planned like orthodontic traction and surgical exposure with orthodontic treatment.

According to the Aktan et al., except for the canine, no tooth type shows a tendency for transmigration in the dental arch [6]. However, Kara et al. had observed two transmigrant cases of lateral incisor and three cases of premolar in their study [14]. In our study we could not find any other tooth involvement for transmigration other than canine. Most of the transmigrant cases are impacted and rarely erupt as was observed in studies conducted by Aktan et al. and Aydin et al. [6, 10]. Only one erupted transmigrated canine (5.0%) was observed in our study as compared to 2.2% of erupted transmigrated canines in a study by Kara et al. [14].

Transmigration is a rare phenomenon with a prevalence of 0.66% in an Indian population. There is an increased prevalence of transmigrant mandibular canines as compared to maxillary canines. The prevalence of transmigrant maxillary canine is almost similar (0.13%–0.20%) in all the studies conducted on transmigration [6, 10, 12]. The panoramic radiographs must be used for evaluation of impacted canines and the usage of CBCT should be implemented in advanced treatment planning as it would provide more precise information about the localisation of transmigrated canine and its relationship to surrounding teeth and median palatine suture. Research studies on transmigration should focus not only on the possible complications like root resorption and follicular spaces but also on the distance beyond the midline migrated by the transmigrated teeth as no literature is yet available. Further research is required to validate that a horizontally impacted maxillary canine is more likely to transmigrate as compared to mesioangularly impacted maxillary canine.

Canine transmigration is of vital significance in dentistry as it can create orthodontic, esthetic, surgical, and interceptive complications. Hereditary patterns of the transmigration should be further investigated as its etiology is not yet ascertained. Type 3 canine is the rarest form of transmigrant mandibular canine and a classification system for maxillary transmigrant canine to aid in the diagnosis and management is long overdue.

## Figures and Tables

**Table 1:** Demographic characteristics of transmigrated teeth.

Documented cases *n* = 3000	Number (prevalence)	Average age (years)	Left side/right side	Males/females	Other developmental dental anomalies presence/absence	Impacted/erupted
Transmigration	20 (0.66%)	24.1	14/6	12/8	9/11	19/1
Mandibular canine transmigration	15 (0.5%)	21.5	11/4	10/5	8/7	14/1
Maxillary canine transmigration	05 (0.16%)	32.0	3/2	2/3	1/4	5/0

**Table 2:** Clinical and radiographic features of transmigrated mandibular canines in 19 patients.

Case	Gender	Age	Transmigrated canine^a^	Type (mandibular canine) Mupparapu's classification	Presence of overretained canine with respect to transmigrated tooth	Other coexisting dental anomalies (except third molars)	Associated pathologies in relation to transmigrated tooth
1	F	17	33	1	No	—	None
2	M	22	33	1	Yes	Agenesis of 12, 22, retained 53, 63	None
3	F	29	43	1	No	Impacted 13, 23, retained 53, 63, 85, agenesis of 45, peg lateral 12, 22	None
4	F	19	33	2	Yes	Retained 53, 63, agenesis of 13, 35, microdont 12, 22	None
5	M	28	33	1	Yes	Retained 73	None
6	F	18	43	5	No	Agenesis of 22, 31, 32, 41	None
7	M	24	33	1	No	—	None
8	M	19	43	1	Yes	Retained 73, 63, missing 12, 22	None
9	M	23	33	1	Yes	—	None
10	M	19	33	1	No	Retained 63	None
11	F	34	13	NA	No	—	None
12	F	19	33	2	No	—	None
13	F	22	23	NA	No	Impacted 13	None
14	M	53	23	NA	No	—	None
15	M	26	33	1	No	—	None
16	M	15	33	4	Yes	Retained 63, impacted 23	FE^*^, mandibular incisors crowding
17	F	18	23	NA	No	—	None
18	M	23	43	4	No	—	FE
19	M	27	33	1	No	—	None
**20**	M	32	13	NA	Yes	—	None

M: male; F: female; ^*^FE: follicular enlargement; NA: not applicable; ^a^tooth numbering in accordance with FDI World Dental Federation notations.

**Table 3:** Characteristics of relationship (position/angulation and distance travelled) between median palatine suture and maxillary transmigrant canine.

Characteristics with median palatine suture	Maxillary transmigrant canines (*n* = 5)
Position and angulation of transmigrated canine	
Horizontal (80°–90°)	4 (80%)
Mesioangular (45°)	1 (20%)
Distance travelled in contralateral arch past midline suture by transmigrated canine along its long axis	
Tip of crown till less than halfway length of crown of transmigrated canine	3 (60%)
Halfway of crown till cementoenamel junction of transmigrated canine	2 (40%)
Beyond cementoenamel junction irrespective of root of transmigrated canine	—

**Table 4:** Summary of published studies on transmigration.

Authors (year)	Number of panoramic radiographs screened	Mandibular canine transmigration prevalence (%)	Maxillary canine transmigration prevalence (%)	Overall canine transmigration prevalence (%)
Mupparapu [9] (2002)	2150	0.004%	—	0.004%
Aydin et al. [10] (2004)	4500	0.18%	0.13%	0.31%
Buyukkurt et al. [11] (2007)	4500	0.33%	—	0.33%
Aras et al. [12] (2008)	6000	—	0.2%	0.2%
Celikoglu et al. [13] (2010)	2215	—	—	0.3%
Gündüz and Çelenk [7] (2010)	12000	—	—	0.1%
Aktan et al. [6] (2010)	5000	0.34%	0.14%	0.48%
Kara et al. [14] (2011)	112873	0.075%	—	0.075%
Halcioglu et al. [15] (2012)	2900	0.06%	0.13%	0.2%
Kumar et al. [8] (2012)	3500	0.46%	—	0.46%
Mazinis et al. [16] (2012)	3586	0.11%	0.06%	0.17%
Kamiloglu and Kelahmet [5] (2014)	453	—	0.44%	0.44%
Present authors (2014)	3000	0.5%	0.16%	0.66%
